# Genome-wide analysis of lectin receptor-like kinases in *Populus*

**DOI:** 10.1186/s12864-016-3026-2

**Published:** 2016-09-01

**Authors:** Yongil Yang, Jessy Labbé, Wellington Muchero, Xiaohan Yang, Sara S. Jawdy, Megan Kennedy, Jenifer Johnson, Avinash Sreedasyam, Jeremy Schmutz, Gerald A. Tuskan, Jin-Gui Chen

**Affiliations:** 1 Biosciences Division, Oak Ridge National Laboratory, Oak Ridge, TN 37831 USA; 2 U.S. Department of Energy Joint Genome Institute, Walnut Creek, CA 94598 USA; 3 HudsonAlpha Institute for Biotechnology, Huntsville, AL 35806 USA

**Keywords:** Lectin domain, Lectin receptor-like kinase (LecRLK), *Populus*, Perennial woody plant, Receptor like-kinase (RLK), Transmembrane kinase

## Abstract

**Background:**

Receptor-like kinases (RLKs) belong to a large protein family with over 600 members in Arabidopsis and over 1000 in rice. Among RLKs, the lectin receptor-like kinases (LecRLKs) possess a characteristic extracellular carbohydrate-binding lectin domain and play important roles in plant development and innate immunity. There are 75 and 173 LecRLKs in Arabidopsis and rice, respectively. However, little is known about LecRLKs in perennial woody plants.

**Results:**

Here we report the genome-wide analysis of classification, domain architecture and expression of LecRLKs in the perennial woody model plant *Populus*. We found that the LecRLK family has expanded in *Populus* to a total of 231, including 180 G-type, 50 L-type and 1 C-type LecRLKs. Expansion of the *Populus* LecRLKs (PtLecRLKs) occurred partially through tandem duplication. Based on domain architecture and orientation features, we classified PtLecRLKs into eight different classes. RNA-seq-based transcriptomics analysis revealed diverse expression patterns of *PtLecRLK* genes among leaves, stems, roots, buds and reproductive tissues and organs.

**Conclusions:**

This study offers a comprehensive view of LecRLKs in the perennial woody model plant *Populus* and provides a foundation for functional characterization of this important family of receptor-like kinases.

**Electronic supplementary material:**

The online version of this article (doi:10.1186/s12864-016-3026-2) contains supplementary material, which is available to authorized users.

## Background

Cell-surface receptors play important roles in perceiving and processing signals at the cellular level. One large family of such cell-surface receptors are the receptor-like kinases (RLKs) [1, 2]. There are over 600 RLKs in Arabidopsis and over 1000 in rice [2]. The role of RLKs as cell-surface receptors perceiving extracellular signals has been validated through functional characterization of several RLK members, e.g., receptor-like Ser/Thr kinases BRASSINOSTEROID-INSENSITIVE 1 [3] and CLAVATA1 [4]. RLKs are typically comprised of an N-terminal extracellular domain, an intermediate transmembrane domain and a C-terminal kinase domain. A total of 15 subfamilies of RLKs have been classified on the basis of their extracellular domains [1].

The lectin receptor-like kinases (LecRLKs) are defined by their characteristic extracellular lectin domain that resembles carbohydrate-binding lectin proteins in humans and animals [5, 6]. However, no LecRLK has been found in the genomes of human or yeast and LecRLKs are viewed as plant-specific [7, 8]. LecRLKs have been best characterized in the two model plant species, Arabidopsis and rice. There are a total of 75 and 173 LecRLKs in Arabidopsis and rice, respectively [8]. Available evidence suggested that *LecRLK* genes are also present in many other plant species including *Nicotiana benthamiana, Solanum lycopersicum, Arabidopsis lyrata, Glycine max, Medicago truncatula, Zea mays* and *Sorghum bicolor* [9–11]. Although the number is low, *LecRLK* genes also exist in the non-vascular and non-seed baring plants, e.g., *Physcomitrella patens* and *Selaginella moellendorffii*, respectively [10]. LecRLKs are further classified into three different forms, i.e., G-type, L-type and C-type, based on the lectin domain identity. There are 32 G-type, 42 L-type and 1 C-type LecRLKs in Arabidopsis, and 100 G-type, 72 L-type and 1 C-type LecRLKs in rice [8]. The G-type LecRLKs were known as B-type LecRLKs due to the resemblance of their extracellular domain with the bulb-lectin proteins in humans and animals [5, 12]. G-type LecRLKs are also known as S-domain RLKs due to the presence of an S-locus domain known to be involved in pollen self-incompatibility [13–16]. The lectin domains of G-type LecRLKs possess a β-barrel structure and are predicted to bind to α-D-mannose. Many G-type LecRLKs also contain a cysteine-rich epidermal growth factor (EGF) domain and a plasminogen/apple/nematode (PAN) domain [8, 17]. The EGF domain is predicted to be involved in the formation of disulfide bonds and the PAN domain is believed to be involved in protein-protein and protein-carbohydrate interactions [18]. It should be noted that EGF and PAN domains are only found in the G-type and are absent in the L-type and C-type LecRLKs. The L-type LecRLKs contain a characteristic legume-lectin domain that is believed to exhibit glucose/mannose-binding specificity [19]; the C-type LecRLKs contain a calcium-dependent carbohydrate-binding lectin domain.

Substantial evidence suggests that LecRLKs play important roles in plant development and innate immunity [17, 20]. However, most studies on LecRLKs were performed in the herbaceous plants. Little is known about LecRLKs in the perennial woody plants which are of significant importance to carbon sequestration, global carbon cycling, environmental and ecological systems and biomass production for forestry and bioenergy industries. *Populus* is a model species for perennial woody plants but there are only a few early studies reporting on the presence of LecRLKs [21–23]. A comprehensive view of LecRLKs in this perennial woody model plant is still lacking. Here we report the genome-wide analysis of classification, domain architecture and expression of LecRLKs in *Populus*.

## Methods

### LecRLK sequence homolog search in *Populus*

*Populus* LecRLK (PtLecRLK) amino acid sequences were collected from v3.0 *Populus trichocarpa* gene annotation curated in the Phytozome (v11.0) database managed by Joint Genome Institute (JGI; www.phytozome.jgi.doe.gov). To identify G-type PtLecRLKs, AT1G65790 (a G-type Arabidopsis LecRLK) was used as a query to collect its *Populus* homologs by dual-affine smith-watermann alignments integrated in Phytozome [24]. We only accepted PtLecRLKs having over 30 % amino acid sequence similarity in the initial alignment. Then, we performed the reciprocal alignment analysis using the *Populus* LecRLK protein (Potri004G028000) showing highest amino acid sequence similarity with AT1G65790 as the input to search for additional potential *Populus* homologs.

The same process was performed to identify L-type and C-type PtLecRLKs using AT2G37710 and AT1G52310 as primary input query, respectively. The L-type PtLecRLK showing highest amino acid sequence similarity with AT2G37710, Potri006G088400, was then used as a template to search for additional potential *Populus* homologs.

In case of isoform information among collected amino acid sequences, the longest full-length amino acid sequences were selected and used for further analyses. These full-length amino acid sequences were subjected to Chromosome Digram module integrated in POPGENIE (popgenie.org) to generate *PtLecRLK* loci location on *Populus* chromosomes [25].

### LecRLK sequence homolog search in moss, shrub, soybean and *Eucalyptus*

To identify LecRLKs in another woody plant, we searched *Eucalyptus grandis* v2.0 genome in phytozome v11.0 by using the same approach that was taken to identify PtLecRLKs. For the identification of G-type LecRLKs in *Eucalyptus*, we performed amino acid sequence alignment using AT1G65790 (a G-type Arabidopsis LecRLK) as the initial query. We collected *Eucalyptus* homologs with over 30 % similarity at the amino acid level with AT1G65790. A second round of protein homolog search was performed by using Potri.004G028000 (a PtLecRLK showing highest amino acid sequence similarity with AT1G65790) as a new input to identify additional potential G-type *Eucalyptus* LecRLKs (EgLecRLKs). Finally, we used Eucgr.D00925, the protein showing highest amino acid similarity with Potri.004G028000, as a template to identify other potential homologs.

To search for L-type and C-type EgLecRLKs, AT2G37710 and AT1G52310 were used as the template, respectively. Then, we used Potri.006G088400 that shows highest amino acid sequence similarity (70 %) with AT2G37710 as a template to identify additional potential homologs of L-type EgLecRLKs. Potri.001G062300, the unique C-type PtLecRLK and the homolog of AT1G52310, was used as a template to confirm the identification of C-type EgLecRLK.

We also extended our search for LecRLKs in moss (*Physcomitrella patens,* v3.3), shrub (*Amborella trichopoda*, v1.0), corn (*Zea mays,* Ensembl-18) and soybean (*Glycine max,* Wm82.a2.v1). We used the same protocol and the same representative *Populus* proteins. Due to the evolutional distance of moss genome, we used 40 % similarity as a threshold to collect the full-length amino acid sequences of moss LecRLKs. For C-type LecRLK analysis, single gene was identified from grape (*Vitis vinifera,* Genescope.12X) genome by the same protocol.

### Functional domain annotation and functional motif prediction of PtLecRLKs

To predict protein functional motifs and domains, including specific lectin and protein kinase domains, the full-length amino acid sequences of PtLecRLKs were subjected to Pfam v29.0 (http://pfam.xfam.org) [26], ScanProsite v20 (http://prosite.expasy.org/scanprosite/) [27] and InterPro v56.0 (https://www.ebi.ac.uk/interpro/) [28] based on HMMER [29]. Since some motifs such as Legume lectin and EGF motif were not predicted in ScanProsite, we merged those annotation results to generate a protein domain structure containing all predicted protein functional domains. From them, we filtered out the protein sequences missing either lectin or kinase domain for further analysis.

To assess the location and number of transmembrane domain (TM), the full-length amino acid sequences used for alignment and phylogenetic analysis were subjected to TMHMM web-based software (v2.0) (www.cbs.dtu.dk/services/TMHMM) [30]. This software also provided the information on membrane transpassing pattern. Significant TM prediction was determined by selecting the probability score ≥ 0.8.

Signal peptide on amino acid sequence was predicted by SignalP v4.0 [31], under a valuable signal sequence selection score ≥ 0.7. When the TM motif was predicted as potential signal peptide, the priority was given to signal peptide prediction.

### Amino acid sequence alignment and phylogenetic analysis

Phylogenetic and amino acid sequence alignment analyses of collected full-length PtLecRLKs were conducted on Geneious R8 software platform (v8.1.2; Biomatters Ltd., New Zealand). The amino acid sequence identity of PtLecRLKs was calculated by ClustalW integrated in Genious R8. ClustalW alignment was run under the typical options composed of BLOSUM 62 cost matrix with the penalty of gap open cost 10 and gap extend cost 0.2. To build phylogenetic tree, the same set of PtLecRLK amino acid sequences were subjected to MUSCLE (v 3.5) by 12 maximum number of iterations together with kmer6_6 for distance measurement under Neighbor-joining clustering method [32]. The best fitting model for each phylogenetic tree construction was performed with MUSCLE alignment result by model selection (ML) method integrated in MEGA7 (v 7.0.18) [33]. We applied the best fitting model for phylogenetic tree by selecting the model having the lowest value of Akaike information criterion (AIC), Bayesian information criterion (BIC), and maximum likelihood (InL) values. To construct the best phylogenetic tree, we performed PHYML method with the best fitting model for each phylogenetic tree [34]. The information of the best fitting model was described in each figure legend of phylogenetic tree. Nearest-Neighbor-Interchange (NNI) was used as a heuristic to improve the likelihood tree.

To validate the phylogenetic tree, we rebuilt another phylogenetic tree by using the neighbor-joining method integrated in Genious software with Jukes-Cantor genetic distance model using bootstrap 1000 replicates resampling to assess statistical strength of phylogenetic tree with the same MUSCLE alignment [33].

### Analysis of gene expression of *PtLecRLKs*

To compare the expression of *PtLecRLKs* in different tissues and developmental stages, we compiled the expressed values of *PtLecRLK* genes from RNA-seq data in different tissues under standard or treatment conditions from the *Populus* Gene Atlas Study at Phytozome (www.phytozome.jgi.doe.gov). Normalized fragments per kilobase of transcript per million mapped reads (FPKM) values were compared to determine gene expression in different tissues. Collected data was summarized by heatmap function in ggplot2 R package [35]. To verify the expression of *PtLecRLKs* identified in the Gene Atlas Study, RT-PCR was performed with gene-specific primers for six *PtLecRLK* genes and the *PtUBCc* as an internal control. All primers used for RT-PCR analysis are listed in the Additional file 1. The same tissues of root tip and root without root tip, internode and node of stem, and young leaf tissue that were originally used for RNA extraction at Oak Ridge National Laboratory for RNA-seq at JGI in the *Populus* Gene Atlas Study were used for RNA extraction for RT-PCR analysis. Total RNA was extracted from adopted tissues with PureLink Plant RNA reagent (Ambion™, Austin, TX) followed by RNA purification with Spectrum Plant RNA purification kit following the manufacturer’s protocol (Sigma-Aldrich, St. Louis, MO). One μg of total RNA was used to synthesize complementary DNA (cDNA) by reverse transcription with Revertaid reverse transcriptase (Thermo Fisher Scientific, Waltham, MA). Ten ng of reversely transcribed cDNA was used to perform PCR reaction with the gene-specific primers in the PCR reaction mixture of DreamTaq Green PCR Master Mix (Thermo Fisher Scientific, Waltham, MA). The PCR reaction was performed with the program consisting of a pre-denaturation at 95 °C for 2 min, 30 cycles of the reaction of 95 °C for 30 s, 57 °C for 30 s and 72 °C for 30 s. Another step of 72 °C for 7 min was followed for the final extension. The PCR amplification results were run on 1 % agarose gel. The gel image was taken by using ChemiDoc XRS+ image analysis system (Bio-Rad, Hercules, CA).

## Results

### *Populus* LecRLKs identification and classification

The full-length amino acid sequences of three representative Arabidopsis LecRLKs (G-type: AT1G65790; L-type: AT2G37710; C-type: AT1G52310) were used initially as templates to search for their sequence homologs encoded by the genome of *Populus trichocarpa* (hereafter refer as *Populus*). To identify additional potential homologs, the *Populus* proteins showing highest amino acid sequence similarity with the corresponding G-, L- and C-type Arabidopsis LecRLKs were then used as templates to search the *Populus* genome again. The search identified a total of 231 unique loci encoding LecRLKs in *Populus* (Table 1). We refined our search criteria to identify only LecRLKs that contain both a lectin domain and a kinase domain. These proteins were then classified as G-type, L-type and C-type PtLecRLKs on the basis of the identity of lectin domain. It should be noted that there were a number of proteins that contain a lectin domain but lacks a kinase domain (Additional file 2). For example, proteins encoded by Potri.010G005900 and Potri.010G017400 contain only a bulb (mannose binding) lectin domain, and as such, did not meet our criteria of being LecRLKs and were not considered in subsequent analyses.

**Table 1:** Number of different types of LecRLKs in Arabidopsis, rice, *Populus*, *Eucalyptus*, shrub, corn, soybean and moss

Plant Species	G-type	L-type	C-type	Total
Arabidopsis	32	42	1	75
Rice	100	72	1	173
*Populus*	180	50	1	231
*Eucalyptus*	118	79	1	198
Shrub	25	30	1	56
Corn	46	48	1	95
Soybean	123	64	2	189
Moss	2	1	2	5

**Fig. 1:**
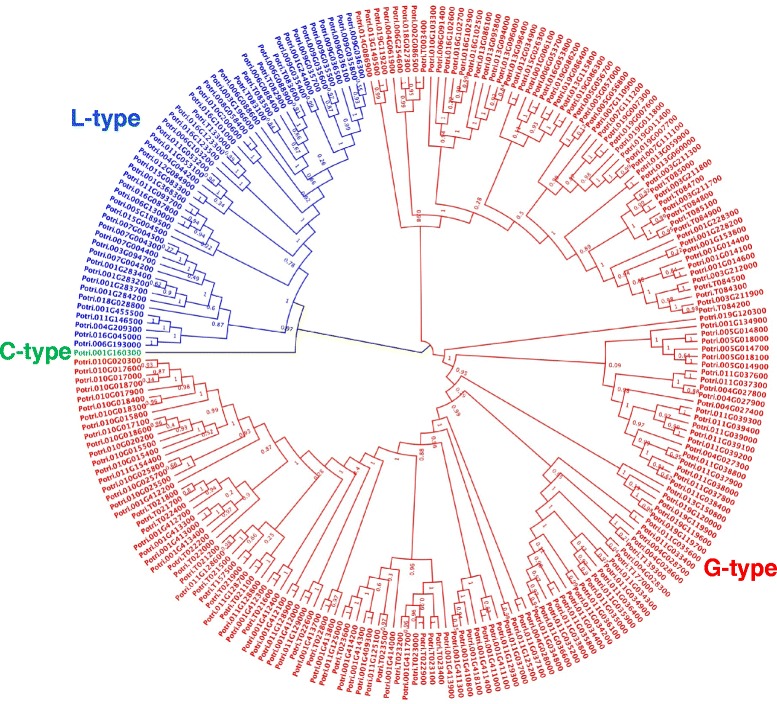
Phylogenetic analysis of full-length amino acid sequences of 231 PtLecRLKs using maximum likelihood tree. The phylogenetic tree was constructed by PHYML with the JTT + G model. aLRT Shimodaira-Hasegawa (SH)-like branch support value is displayed in each node. Note that three different types of PtLecRLKs are categorized clearly in three different clades (blue: L-type; green: C-type; red: G-type)

In order to validate the classification of PtLecRLKs into alternate types, we performed three independent analyses using full-length amino acid sequences; i.e., i) amino acid sequence alignment; ii) amino acid identity and iii) phylogenetic analysis. We first analyzed randomly selected proteins, three each from G- and L-type, plus the sole C-type PtLecRLK. As shown in the Additional file 3, the lectin domain regions were distinct from each other whereas the protein kinase domains were highly conserved. The phylogenetic analysis with full-length amino acid sequences revealed that members from each type formed separate clades, with the L-type PtLecRLKs showing at least 47 % amino acid identity each other and the G-type showing at least 66 % amino acid identity between currently selected proteins (Additional file 3). On the basis of these results, phylogenetic analysis was performed with full-length amino acid sequences of all 231 PtLecRLKs. As shown in Fig. 1, three different types of PtLecRLKs were clearly separated in three different clades.

In total, 231 PtLecRLKs with 180 G-type, 50 L-type and 1 C-type were identified (Table 1; Additional files 4, 5 and 6). The total number of LecRLKs in *Populus* (231) is about three times that in Arabidopsis (Table 1). Comparison between the numbers of LecRLKs in these two dicot species also revealed that the number of G-type LecRLKs in *Populus* is larger than L-type (G-type: 180 vs L-type: 50) whereas in Arabidopsis [8], there are more L-type than G-type LecRLKs (G-type: 32 vs L-type: 42). Interestingly, there are also more G-type than L-type LecRLKs in rice (G-type: 100 vs L-type: 72), similar to that in *Populus*. However, although the total number of LecRLKs in *Populus* is larger than that in rice, *Populus* has fewer L-type LecRLKs than rice (Table 1).

To extend our analysis to other woody species, we performed genome-wide search for LecRLKs in the *Eucalyptus* genome by the same approach that we used for identifying PtLecRLKs. In addition, we searched LecRLK distribution in four more species including moss, shrub, corn and soybean. In total, we identified 198 EgLecRLKs (G-type: 118, L-type: 79, C-type: 1) (Table 1; Additional file 7). Soybean has 189 LecRLKs (G-type: 123, L-type: 64, C-type: 2). Similar to *Populus*, *Eucalyptus* and soybean also have more G-type LecRLKs than L-type. In shrub and corn, a total of 56 (G-type: 25, L-type: 30, C-type: 1) and 95 (G-type: 46, L-type: 48, C-type: 1), respectively, were identified. Moss has only 5 LecRLKs. All tested genomes have one or two C-type LecRLK (Table 1; Additional file 7). Taken together, these results suggest that the G-type LecRLKs have been disproportionately expanded in *Populus*, compared to Arabidopsis. On the other hand, *Populus* contains only one C-type LecRLK, identical to that in Arabidopsis, rice and *Eucalyptus*.

### Tandem repeats and size difference of PtLecRLKs

**Fig. 2:**
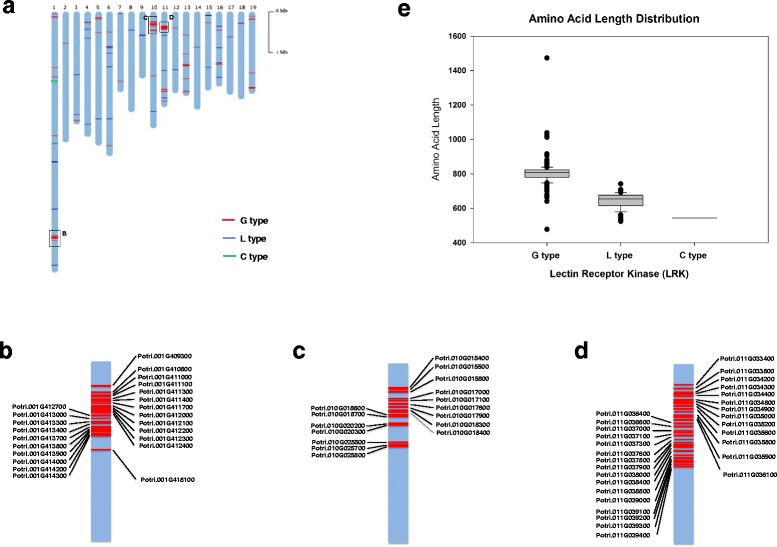
Chromosomal distribution of *PtLecRLK* genes in the genome of *Populus trichocarpa.* **a** Chromosomal location of G-type (shown in red), L-type (shown in blue) and C-type (shown in green) *LecRLK* genes in the *Populus* genome. Note that 180 G-type *PtLecRLK* genes are distributed in 16 *Populus* chromosomes (not in chromosomes 8, 9 and 17). Super tandem repeat regions are boxed in chromosomes 1, 10 and 11. **b** Super tandem repeats of G-type *PtLecRLK* genes in chromosome 1. **c** Super tandem repeats of G-type *PtLecRLK* genes in chromosome 10. **d** Super tandem repeats of G-type *PtLecRLK* genes in chromosome 11. **e** Predicted amino acid number of three different types of PtLecRLKs

Among 231 PtLecRLKs, a total of 195 PtLecRLK loci were distributed across most *Populus* chromosomes; 36 *PtLecRLK* genes were annotated on scaffolds with an indeterminate chromosomal location. Of the 195 *PtLecRLK* genes, approximately 38 % of the G-type *PtLecRLK*s were found as clusters of tandem repeats (Fig. 2a). In some chromosomal locations on chromosome 1, 10, and 11, there were more than 20 *PtLecRLK* genes clustered together (named “super tandem repeat region” here) (Fig. 2b-d). This tandem repeat feature is similar to that reported in *Brassicaceae* and related outgroups [36] and is likely a major attributor for the G-type LecRLK family expansion.

**Fig. 3:**
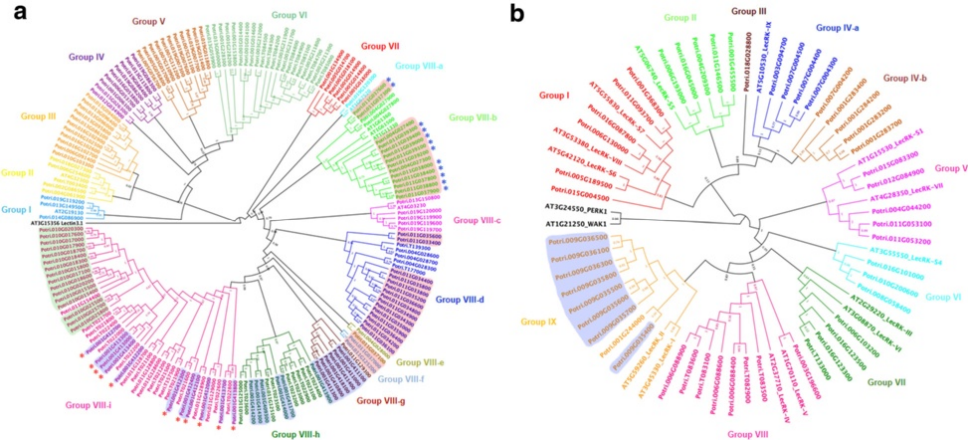
Classification of different groups of G- and L-type PtLecRLKs based on the phylogenetic analysis using maximum likelihood method (PHYML). The full-length amino acid sequences of PtLecRLKs were aligned using MUSCLE. The maximum likelihood phylogenetic tree was constructed by PHYML. aLRT SH-like branch support value is displayed in each node. **a** Phylogenetic tree of G-type PtLecRLKs. Maximum likelihood tree was calculated with Le Gascuel (LG) + G model with 180 G-type PtLecRLKs and 8 representative G-type AtLecRLKs. AT3G15356 (Lectin 3.1) was rooted to build phylogenetic tree. Three super tandem repeated regions shown in Fig. 2 are highlighted (light blue: super tandem repeats of *PtLecRLK* genes in chromosome 1; light green: super tandem repeats of *PtLecRLK* genes in chromosome 10; light red: super tandem repeats of *PtLecRLK* genes in chromosome 11). The loci in alternate clade among tandem repeat *PtLecRLK* genes on chromosome 11 are marked with blue asterisks. The loci in alternate clade in tandem repeat *PtLecRLK* genes on chromosome 1 are labeled with red asterisks. **b** Phylogenetic tree of L-type PtLecRLKs. Maximum likelihood tree was calculated with LG + G model with 50 L-type PtLecRLKs and 14 AtLecRLKs. The distantly related AT1G21250 (WAK1) and AT3G24550 (PERK1) were used as distal proteins to construct phylogenetic tree. A tandem repeated region is highlighted by light blue

Maximum likelihood tree of G-type PtLecRLKs showed that tandem repeat genes on chromosome 10 were clustered in the same clade (Fig. 3a). PtLecRLK tandem repeat genes in the super tandem repeat regions of chromosome 1 were clustered in several neighboring clades (Fig. 3a). Twelve out of 29 tandem repeat genes on chromosome 11 also occurred in an alternate clade that was clearly separated from the clade with the rest of genes (light blue: chromosome 1, light green: chromosome 10, and light red: chromosome 11; Fig. 3a). Unlike G-type PtLecRLKs, the only tandem repeat region for L-type PtLecRLKs was found in the chromosome 9 where a total of 8 L-type PtLecRLKs were clustered together (Fig. 3b).

By examining amino acid number in each PtLecRLK, we found that G-type PtLecRLKs are generally larger than L-type and C-type, with the average of 806 amino acids versus 645 in L-type and 544 in C-type (Fig. 2e). This is largely due to the fact that in addition to the lectin domain, G-type PtLecRLKs often contain the EGF and PAN domains as well as the S-locus glycoprotein domain. In addition, some G-type PtLecRLKs contain a DUF3403 domain in the C-terminus, posterior to the kinase domain. The largest PtLecRLK (Potri.T084700) has 1473 amino acids.

### Phylogenetic analysis of PtLecRLKs

To perform the phylogenetic tree analysis, maximum likelihood tree was constructed from alignment result using full-length amino acid sequences of PtLecRLKs. For G-type phylogenetic tree construction, an Arabidopsis lectin 3.1 (AT3G15356; L-type LecRLK) was used as an unrelated protein. This protein was also used as an outgroup in the previous phylogenetic analysis of Arabidopsis and rice G-type LecRLKs [8]. In addition, 8 G-type Arabidopsis LecRLKs that were classified as the representative genes in each group in phylogenetic tree [8] were subjected together with 180 G-type PtLecRLKs to build a phylogenetic tree. The phylogenetic tree of G-type PtLecRLKs was constructed and clustered into several groups. The grouping was done in a manner similar to what was done for Arabidopsis and rice LecRLKs [8]. These 180 G-type PtLecRLKs were divided into 8 large cluster groups (Fig. 3a). Based on distinct clade formation, the group VIII was subdivided into 9 different subgroups (VIII-a to VIII-i). All those super tandem repeated PtLecRLKs were categorized in group VIII as highlighted by different color (light blue: chromosome 1, light green: chromosome 10, and light red: chromosome 11; Fig. 3a). Group III to VII clades did not contain any Arabidopsis G-type LecRLKs.

**Fig. 4:**
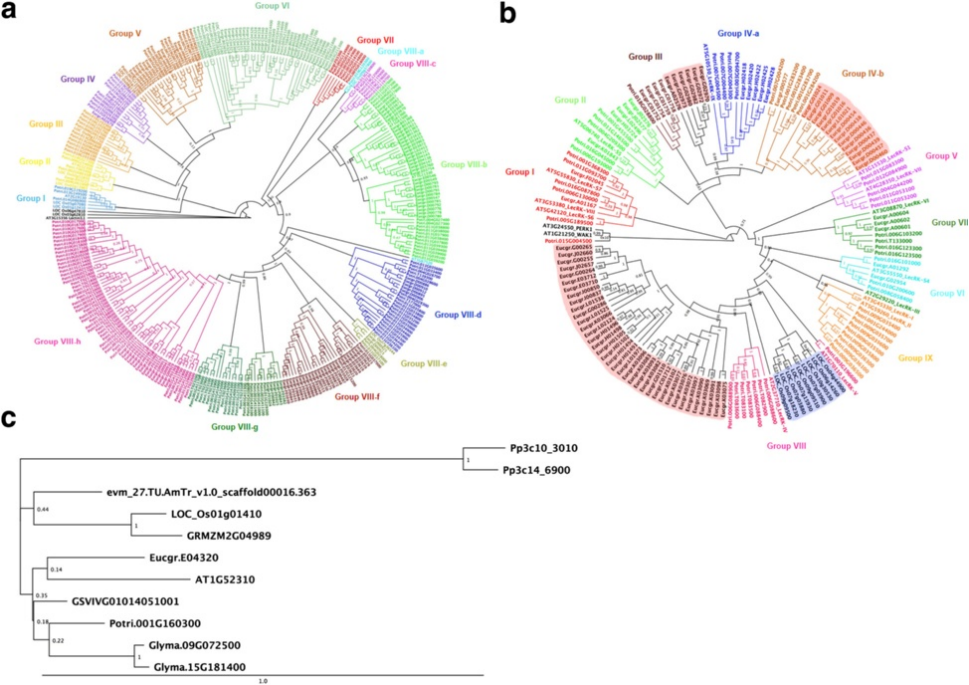
Maximum likelihood phylogenetic analysis of G-, L-, and C-type PtLecRLKs with LecRLKs from Arabidopsis, rice and *Eucalyptus*. The full-length amino acid sequences of G- and L-type LecRLKs were collected from Arabidopsis, rice, and *Eucalyptus* genome to perform phylogeny analysis with those of *Populus*. C-type PtLecRLK was tested with full length amino acid sequences of those of moss (*Physcomitrella patens*), shrub (*Amborella trichopoda*), corn (*Zea mays*), soybean (*Glycine max*) and grape (*Vitis vinifera*) as well as those of Arabidopsis, rice and *Eucalyptus*. **a** Maximum likelihood for constructing G-type LecRLKs phylogenetic tree calculated by LG + G model. aLRT branch support is displayed in each node. AT3G15356 (Lectin 3.1) was used as a distal protein to build phylogenetic tree. **b** Maximum likelihood phylogenetic tree with JTT + G + invariant sites (I) model of L-type LecRLKs. The distantly related AT1G21250 (WAK1) and AT3G24550 (PERK1) were rooted to classify groups in this analysis. aLRT branch support value is displayed in each node. The clades including only G-type EgLecRLKs are highlighted with light red. A clade including G-type rice LecRLK is highlighted with light blue. **c** Maximum likelihood phylogenetic tree of C-type LecRLKs with JTT + G model using 1000 bootstrap. Bootstrap values are shown to each node. The bar indicates the number of amino acids substitution per site

Through the same process, 50 L-type PtLecRLKs were subjected to phylogenetic analysis. Two distant Arabidopsis proteins, PEPK1 and WAK1, and 14 Arabidopsis L-type LecRLKs that were reported as the representative L-type AtLecRLKs in each group were used to define each clade [8, 37]. Based on the full-length amino acid sequence alignment of L-type PtLecRLKs with Arabidopsis proteins, the maximum likelihood phylogenetic tree showed nine major groups that are classified by different clade separation (Fig. 3b). Group IV was divided into two subgroups. This is also supported by the phylogenetic analysis of additional L-type LecRLKs from *Eucalyptus* (Fig. 4b). Each group clade, except group III of singleton clade, contains at least one Arabidopsis L-type LecRLK (Fig. 3b).

The constructed phylogenetic trees of G- and L-type PtLecRLKs were validated by a different phylogenetic method using neighbor joining with 1000 bootstraps (Additional file 8). Most clade formations were similar between these two methods except minor differences in clustering. For example, group VIII-f merged into group VIII-h clade in neighbor joining phylogenetic tree (Additional file 8). Nonetheless, PtLecRLKs were consistently formatted into the same clade by using these two different methods for phylogenetic tree construction (Fig. 3, Additional file 8).

To assess whether the PtLecRLKs were evolutionally separated from LecRLKs in a different woody plant species, we compiled LecRLKs from *Eucalyptus grandis* v2.0 genome in Phytozyme v11.0. *Eucalyptus* genome has a total of 198 EgLecRLKs (118 G-type, 79 L-type, and 1 C-type). For G-type LecRLK amino acid sequence alignment and phylogeny analysis, 180 G-type PtLecRLK were analyzed together with 118 G-type EgLecRLKs, 8 representative Arabidopsis LecRLKs [8] including a distant Arabidopsis protein (AtLec3.1), and 13 rice representative G-type LecRLKs that were used as the representative members in each group to classify rice G-type LecRLKs [8]. Although we included *Eucalyptus* and rice LecRLKs in the analysis, typical nodes or branches shown in this tree (Fig. 4a) is similar to the tree constructed using *Populus* and Arabidopsis LecRLKs (Fig. 3a). The groups VII and VIIIf-h clades were only shared by G-type LecRLKs from woody plants without LecRLKs from herbaceous plants (Fig. 4a). This observation was consistent with the phylogenetic trees built by a different tree building method (Geneious tree builder) using neighbor joining method with 1000 bootstrap resampling (Additional file 9). This phylogenetic tree showed that three genes of group II were divided into different new clades. Group VI was also divided into two different clades. Except for these two minor differences, the clustering and grouping results were almost identical between these two methods for phylogenetic tree construction.

Same analysis was performed for L-type LecRLKs by using all 50 PtLecRLKs together with all 79 EgLecRLKs, 14 AtLecRLKs identified as the representative genes of each group in the published study [37], and 9 rice LecRLKs identified as the representative genes of each group in rice L-type LecRLK analysis in the published study [8]. The maximum likelihood phylogenetic tree showed that the singleton clade of Group III shared its clade with four G-type EgLecRLKs (Fig. 4b). Group IV-b was also divided to two clades with EgLecRLKs (Fig. 4b). All nine representative rice L-type LecRLKs were integrated into group VIII. This clade was distinctly separated from L-type LecRLKs from other species (highlighted by light blue; Fig. 4b). The clade next to group VIII was the largest cluster consisting of only EgLecRLKs (Fig. 4b). In total, three distinct clades were associated with EgLecRLKs only (highlighted by light red; Fig. 4b). Again, this pattern was also observed in the tree constructed by using the neighbor joining with 1,000 bootstrap resampling method (Additional file 9).

In the case of C-type LecRLK, we identified single C-type LecRLK in *Populus*, *Eucalyptus*, Arabidopsis and rice genomes by amino acid sequence alignment with Arabidopsis C-type LecRLK. To examine whether C-type LecRLK is also present as a single copy in other species, we extended our search to moss (*Physcomitrella patens*), shrub (*Amborella trichopoda*), corn (*Zea mays*), soybean (*Glycine max*) and grape (*Vitis vinifera*). Single copy of C-type LecRLK was identified in the grape, corn and shrub genomes whereas moss and soybean had two C-type LecRLKs. To examine the evolutional relationship of C-type LecRLK, we constructed phylogenetic tree with full-length amino acid sequences of C-type LecRLKs. The clade containing moss C-type LecRLKs was placed far away from other land plant species. C-type LecRLKs of shrub is closer with that of monocot plant than dicot woody and herbaceous plants (Fig. 4c). The phylogenetic tree using neighbor-joining method also showed a similar pattern (Additional file 9).

### Domain architecture of PtLecRLKs

**Fig. 5:**
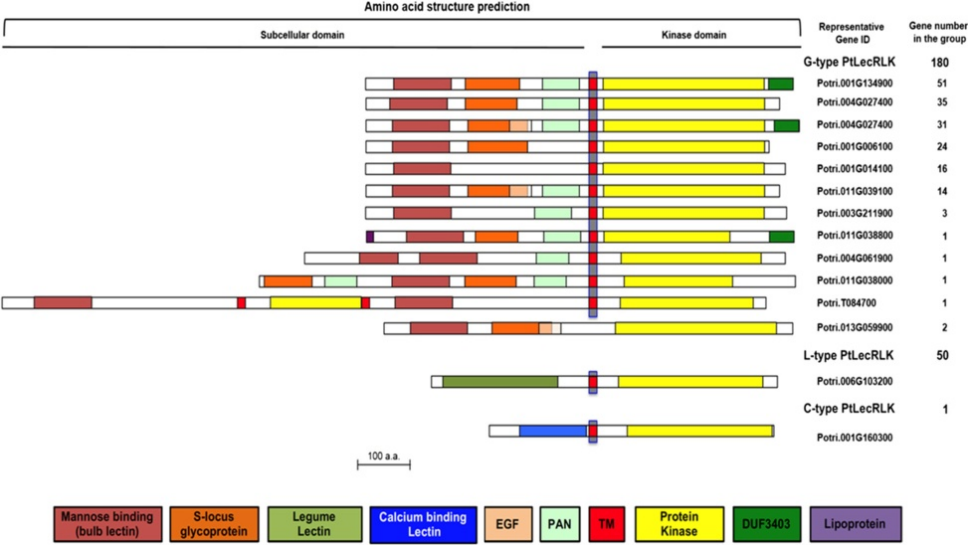
Domain architecture of PtLecRLKs. G-type PtLecRLKs (total 180) generally consist of bulb lectin (mannose binding) domain and S-locus glycoprotein domain at the N-terminus and protein kinase domain at the C-terminus, separated by the transmembrane domain. Some G-type PtLecRLKs also contain EGF, PAN and DUF3404 domains. L-type PtLecRLKs (total 50) contain the extracellular legume lectin domain. C-type PtLecRLK contains the calcium-binding lectin domain

As noted above, G-type PtLecRLKs often contain mannose binding bulb-lectin domain, S-locus glycoprotein domain, EGF domain, PAN domain and DUF3403 domain and thus having more diverse domain architectures. Within G-type, there are 31 PtLecRLKs that contain all these five domains; only 16 G-type PtLecRLKs contain a single domain, the bulb-lectin domain (Fig. 5). A total of 159 out of 180 G-type PtLecRLKs contain the S-locus glycoprotein domain. Some unique domain architectures were also observed within the G-type PtLecRLKs. For example, one G-type PtLecRLK (Potri.004G061900) contains two tandem bulb-lectin domains. Another G-type PtLecRLK (Potri.011G038000) contains two S-locus protein domains and two PAN domains (Fig. 5). The largest G-type PtLecRLK (Potri.T084700) appears to be a fusion protein of two PtLecRLKs with two bulb-lectin domains and two kinase domains (Fig. 5). A prokaryotic lipoprotein lipid attachment motif was identified in a G-type PtLecRLK, Potri.011G03880 (Fig. 5), implying that this protein is potentially subjected to post-translational modification for membrane localization. A total of 83 G-type PtLecRLKs contain the DUF3403 domain at their C-terminus, posterior to the protein kinase domain (Fig. 5). This DUF3403 domain has also been reported in LecRLKs from other plant species [10, 38] but its function is unknown.

Four G-type PtLecRLKs have the truncated lectin domain, lacking at least 10 amino acids, and 7 other G-type PtLecRLKs have a truncated protein kinase domain (Additional file 10). In addition, we identified three highly conserved motifs in the bulb lectin domain and two cysteine-rich motifs (C-rich), localized in the C-terminal region of EGF motif and the middle of PAN domain (Additional file 10).

Compared with G-type, the domain architecture of L-type and C-type PtLecRLKs are less complex (Fig. 5). All L-type PtLecRLKs contain a single legume lectin domain. However, six L-type PtLecRLKs had a truncated legume lectin domain and 4 others lack middle portion of conserved protein kinase domain (Additional file 11). In addition, we identified three highly conserved motifs in the legume lectin domain of L-type PtLecRLKs (Additional file 11).

### Domain orientation of PtLecRLKs

**Fig. 6:**
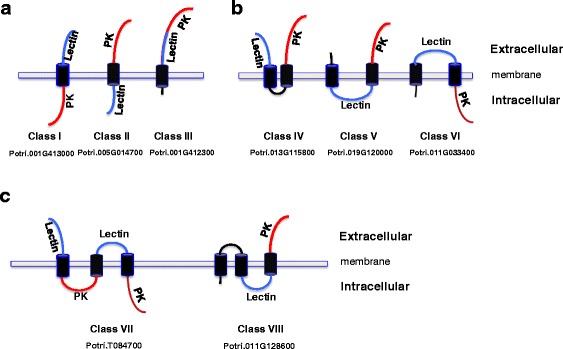
Domain orientation of PtLecRLKs. On the basis of prediction of number of transmembrane domain (TM) and the orientation of the lectin domain and the protein kinase domain, PtLecRLKs were categorized into 8 different classes. **a** Categorization of single-pass transmembrane PtLecRLKs (Class I, II and III). **b** Categorization of double-pass transmembrane PtLecRLKs (Class IV, V and VI). **c** Categorization of triple-pass transmembrane PtLecRLKs (Class VII and VIII). Examples of representative member of each class are shown below each class

RLKs are typically comprised of an extracellular domain, a TM and an intracellular kinase domain. Our analysis of the TM domain, however, has revealed several interesting features for PtLecRLKs. We identified PtLecRLKs with diverse domain orientation features and classified them into eight different classes based on computational approaches of Krogh et al. [30]. These eight different classes were first grouped on the basis of the number of TM domains, and then further separated by the position of lectin domain and kinase domain (Fig. 6). Class I to III PtLecRLKs have one, Class IV to Class VI have two, and Class VII and Class VIII have three TM domains. Class I PtLecRLKs have a typical extracellular lectin domain and an intracellular kinase domain (i.e., Potri.001G41300) (Fig. 6); there are 24 G-type and 15 L-type PtLecRLKs falling into the Class I category (Table 2). Class II G-type PtLecRLKs have a reversed extracellular kinase domain and an intracellular lectin domain (i.e., Potri.005G014700), opposite of Class I; there are 100 G-type proteins in this category, representing the largest group of G-type PtLecRLKs. Class III PtLecRLKs have an extracellular lectin domain and an extracellular kinase domain (i.e., Potri.001G412300). Class IV PtLecRLKs also have both extracellular lectin domain and extracellular kinase domain but these domains are separated by two inverted TM domains (i.e., Potri.013G115800). Class V PtLecRLKs have an extracellular kinase domain and an intracellular lectin domain with two inverted TM domains (i.e., Potri.019G120000). Class VI PtLecRLKs have an extracellular lectin domain and an intracellular kinase domain with two inverted TM domains (i.e., Potri.011G033400), opposite of Class V. Class VII PtLecRLK has two extracellular lectin domains and two kinase domains with three TM domains (i.e., Potri.T084700), resembling the fusion of two Class I PtLecRLKs. Class VIII PtLecRLK has an intracellular lectin domain and an extracellular kinase domain with three TM domains (i.e., Potri.011G128600). Class VII and VIII PtLecRLKs (with three TM domains) were only identified in G-type PtLecRLKs. It should be noted that such unusual domain orientation has been reported in LecRLKs in other plant species [36], but appeared to be more abundant in *Populus*.

**Table 2:** Number of PtLecRLKs in different classes. The classification was based on the predictions of number of transmembrane domain and the orientation of the lectin domain and the protein kinase domain. Only proteins with predicted transmembrane domain are included in this analysis

Class	Number of transmembrane	Position of lectin domain	Position of kinase domain	G-type	L-type	C-type	Total
I	1	extracellular	intracellular	24	15	0	39
II	1	intracellular	extracellular	100	14	1	115
III	1	extracellular	extracellular	1	1	0	2
IV	2	extracellular	extracellular	7	4	0	11
V	2	intracellular	extracellular	3	3	0	6
VI	2	extracellular	intracellular	10	1	0	11
VII	3	extracellular	intracellular	1	0	0	1
VIII	3	intracellular	extracellular	1	0	0	1

Although L-type PtLecRLKs have much simpler domain architecture than G-type, 8 out of 50 L-type PtLecRLKs contain two TM domains (Table 2; Additional file 5). Twenty-nine L-type PtLecRLKs were grouped in Class I and Class II (Table 2; Additional file 5). The single C-type PtLecRLK was in Class II (Table 2; Additional file 6). It should be noted that not all PtLecRLKs were predicted to contain the TM domain. TM was not found in 33 G-type and 12 L-type PtLecRLKs (Additional files 4, 5, 10 and 11). The complete domain architecture and orientation of G-, L- and C-type PtLecRLKs are listed in Additional files 4, 5 and 6, respectively.

### Expression patterns of *PtLecRLK* genes

**Fig. 7:**
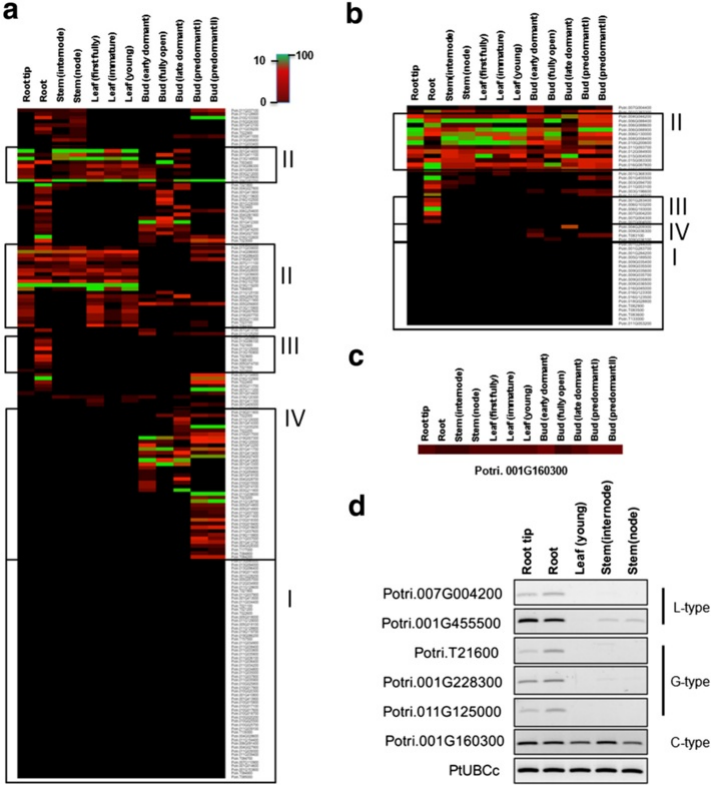
Expression patterns of *PtLecRLK* genes. RNA-seq data were collected from the *Populus* Gene Atlas Study in Phytozome v11.0 (http://phytozome.jgi.doe.gov/pz/portal.html). The FPKM value of four different tissue types including root (root ant root tip), leaf (immature, young and floral), stem (internode and node) and bud (predormant stage I and II, early and late dormant, and fully opened) were analyzed. The tissue specificity of gene expression was determined by the comparison of FPKM value ≥1 in a given tissue versus FPKM < 1 in other tissues. Four different zones were categorized (Zone I: transcript undetectable in all tissues; Zone II: transcript detected in all tissue types; Zone III: transcript only detected in roots; Zone IV: transcript only detected in bud tissues). **a** The expression patterns of the G-type *PtLecRLK* genes. **b** The expression patterns of the L-type *PtLecRLK* genes. **c** The expression pattern of the C-type *PtLecRLK* gene. **d** RT-PCR analysis

Little is known about the function of LecRLKs in *Populus*. As the first attempt to provide insights into their potential functions, we analyzed the expression of *PtLecRLK* genes across various tissues and organs by mining RNA-seq data from the *Populus* Gene Atlas Study in Phytozome v11.0 (http://phytozome.jgi.doe.gov). In this study, there are 24 different samples including samples collected under standard and treatment conditions. We complied the FPKM values of *PtLecRLK* genes in three different types detected in 24 different samples in the Additional files 12, 13 and 14. We also generated heatmap image of 231 *PtLecRLK* genes collected from 12 different samples under standard conditions (Fig. 7). These datasets contain four different tissue types of different growth stages including two root samples (root and root tip), three leaf samples (immature, first fully expanded and young), two stem samples (internode and node), and five bud samples (early dormant, fully open, late dormant, predormant I and predormant II stage). A large number of *PtLecRLK* genes (G-type: 59; L-type: 19) showed low (FPKM < 1) or undetectable expression (FPKM = 0) in the interrogated tissues (Fig. 7, designated zone I). A total of 28 *PtLecRLK* genes (G-type: 13, L-type: 14, and C-type: 1) showed expression across all tissues (Fig. 7, zone II). A total of 22 *PtLecRLK* genes (G-type: 16; L-type: 6) showed root-specific expression (Fig. 7, zone III) (Additional file 15). The bud tissues had the largest number of tissue-specific *PtLecRLK* genes with 41 G-type and 4 L-type (Fig. 7, zone IV; Table 3). Three genes including Potri.011G033400, Potri.013G095800 and Potri.010G015400 were expressed solely in the stem tissues. A G-type *PtLecRLK* gene was expressed specifically in the leaf tissues with FPKM value close to 1 (Table 3; Additional file 12). In the analysis of reproductive tissues/organs from female and male *Populus* trees, we observed that 21 G-type and 9 L-type *PtLecRLK* genes were specifically expressed in female reproductive tissues/organs. Nine G-type and 1 L-type *PtLecRLK* genes were expressed only in male reproductive tissues/organs (Additional files 16 and 17). The sole C-type *PtLecRLK* gene was expressed across all tissues examined (Fig. 7c; Additional file 14). The distribution of the number of *PtLecRLK* genes from each group expressed in different tissues and organs is summarized in the Additional file 18. In general, no any group of *PtLecRLK* genes was uniquely expressed in a given tissue or organ.

**Table 3:** Number of *PtLecRLK* genes specifically expressed in root, stem, leaf or bud tissues

	Root	Stem	Leaf	Bud	In all four tissues	In two tissues^a^	In three tissues^b^	Low or no expression^c^	Total
G-type	16	3	1	41	13	29	18	59	180
L-type	6	0	0	4	14	5	2	19	50
C-type	0	0	0	0	1	0	0	0	1
Total	22	3	1	45	28	34	20	78	231

^a^In two tissues: only expressed in two tissues but not in other tissues. FPKM cutoff value = 1

^b^In three tissues: only expressed in three tissues but not in the other tissue

^c^Low or no expression: FPKM = 0 or <1

To verify the Gene Atlas dataset and tissue-specific gene expression in the tested tissues, we performed RT-PCR analysis with gene-specific primers using cDNA generated from two root samples, one leaf sample, and two stem samples that were used for the *Populus* Gene Atlas Study. Two genes from L-type and three genes from G-type, which were detected with higher FPKM value in root tissue than other tissues, were tested together with the sole C-type *PtLecRLK*. Consistent with the RNA-seq data, C-type *PtLecRLK* was detected across roots, leaf and stems (Fig. 7d). Potr.007G004200, an L-type *PtLecRLK*, was detected in root tissues only. The other L-type *PtLecRLK*, Potri.001G455500, was detected in root tissues and also weakly in two stem tissues (Fig. 7d). No transcript of these two L-type *PtLecRLK* genes was detected in young leaf tissues, which is consistent with Gene Atlas dataset (Additional file 13). For G-type *PtLecRLKs*, three tested genes were only detected in the root tissues (Fig. 7d). Taken together, the results from the RT-PCR analysis was largely consistent with the Gene Atlas data.

## Discussion

The membrane-bound LecRLKs are believed to play important roles in the cellular responses to external stimuli including pathogen attack, environmental stress and developmental clues [17, 20]. Current literatures reporting the classification and function of LecRLKs have been limited in herbaceous plant including Arabidopsis, tobacco, rice and tomato [8, 36, 37]. In this study, we identified 180 G-type, 50 L-type and 1 C-type LecRLKs in the perennial woody model plant *Populus trichocarpa*. Our studies revealed several important features of LecRLKs in *Populus*.

### Expansion of LecRLKs in *Populus*

Comparison of the total number of different types of LecRLKs among Arabidopsis, rice, corn, shrub, soybean, *Eucalyptus* and *Populus* revealed that the number of G-type LecRLKs differed drastically among these species (Table 1), and that in *Populus*, G-type LecRLKs have expanded. This notion is supported by the analysis of LecRLKs in another woody species, *Eucalyptus*. We identified a total of 118 G-type EgLecRLK out of 198 EgLecRLKs (Table 1; Additional file 7). Moreover, it was previously reported that the L-type LecRLKs were expanded than G-type LecRLKs in Arabidopsis [37]. However, in *Populus*, the number of G-type LecRLKs is over three times that of L-type LecRLKs (Table 1). Because the majority (>88 %) of G-type PtLecRLKs contains the S-locus glycoprotein domain which is an essential domain for sporophytic self-incompatibility response-related proteins, the difference in the number of G-type LecRLKs between Arabidopsis (G-type: 32 vs L-type: 42) and *Populus* (G-type: 180 vs L-type: 50) may be partially explained by the fact that Arabidopsis is a self-fertile plant whereas *Populus* is an obligate outcrossing plant. Because LecRLKs are predominantly hypothesized to participate in biotic stress tolerance [12, 37, 38], the large number of *LecRLKs* in *Populus* may also suggest that perennial plants have evolved a large array of LecRLKs in response to longer life cycles and larger probability of exposure to more diverse microbial populations.

Based on phylogenetic analysis of full-length amino acid sequences of LecRLKs from *Populus*, Arabidopsis, *Eucalyptus* and rice, both G-type and L-type LecRLKs have the divergent group only associated with two woody species, implicating that woody plant LecRLKs may have evolved divergent functions than herbaceous plants (Fig. 4; Additional file 9). Interestingly, a major part of *Eucalyptus* L-type LecRLKs formed a unique clade next to PtLecRLKs (Fig. 4b; Additional file 9).

A total of 56 G-type *PtLecRLK* genes were expressed in the reproductive tissues/organs of female and male *Populus* plant (Additional file 17). Among them, 21 G-type *PtLecRLK* genes were specifically expressed in female reproductive tissues/organs whereas 9 G-type *PtLecRLK* genes were specifically expressed in the male. It should be noted that the S-locus domain localized typically on G-type LecRLKs was initially reported as the pollen factor inducing the rejection of self-pollen in the self-incompatibility process [39]. Since *Populus* is a dioecious species, S-locus domain-containing LecRLKs are likely to have evolved new functions beyond its identified function in self-incompatibility.

### Tandem repeats of PtLecRLKs

A total of 195 out of 231 *PtLecRLK* loci were assigned with chromosomal location and were distributed on most *Populus* chromosomes. Similar to what has been reported in other plant genomes [36, 37], *PtLecRLK*s were found in tandem repeats in many chromosomes. Three super tandem repeat regions of G-type *PtLecRLK* genes were identified in chromosomes 1, 10 and 11 (Fig. 2). These super tandem duplicate gene clusters generally have high phylogenetic proximity each other as shown in the phylogenetic analysis (Fig. 3a). Interestingly, the super tandem repeat genes of G-type *PtLecRLKs* in chromosome 11 and 1 were less strictly clustered, implying that these *PtLecRLK*s may have evolved different functions. These tandem duplicated genes do not appear to be derived from the Salicoid whole-genome duplication event [40], such that, the super tandem repeat region identified in the chromosome 10 (all G-type PtLecRLKs in this region) was absent on the chromosome 8 (Fig. 2).

### Domain architecture and organization of PtLecRLKs

By analyzing the predictions of domain architecture and organization, we observed several interesting features of PtLecRLKs. Firstly, in addition to the bulb lectin domain, G-type PtLecRLKs often contain other domains including S-locus glycoprotein domain, EGF domain, PAN domain and DUF3403 domain. A total of 31 G-type PtLecRLKs contain all of these five domains. The DUF3403 domain was found in 83 G-type PtLecRLKs at their C-terminus posterior to the protein kinase domain (Fig. 5). LecRLKs are typically composed of an extracellular lectin domain and an intracellular PK domain, and in our study, we were able to classify PtLecRLKs into 8 classes on the basis of TM domain predictions and found that most PtLecRLKs belongs to the single TM protein group (Classes I, II and II), thereby functioning as potential membrane bound receptors. However, we also identified a number of PtLecRLKs with two or three TM domains (Fig. 6 and Table 2). Surprisingly, unlike reports from herbaceous plants, there are more PtLecRLKs predicted to have an intracellular lectin domain and an extracellular protein kinase domain than PtLecRLKs with an extracellular lectin domain and an intracellular protein kinase domain (Table 2). The functional significance of these observations remains undefined.

Two cysteine-rich motifs were identified in the C-terminal region of EGF motif and the middle of PAN domain of G-type PtLecRLKs (Additional file 10). These motifs may serve as potential protein-protein interaction sites. A number of PtLecRLKs did not contain the TM domain (Additional files 4 and 5). Therefore, the action of such proteins may not be restricted to signal detection at the cell surface and may function in a non-membrane bound context. Finally, a number of proteins with bulb lectin domain and legume lectin domain, but without the kinase domain, were identified (Additional files 2 and 19). Interestingly, some of these proteins also contain the EGF, PAN or DUF3403 domain (Additional files 2 and 19). The transcript of genes encoding these lectin domain-containing proteins were detected in the *Populus* Gene Atlas (data not shown), suggesting that they are functional. Presumably, these lectin domain-containing proteins can still bind carbohydrate ligands but their downstream actions do not rely on phosphorylation activity.

### Functional implication of PtLecRLKs

LecRLKs are specifically present in the plant kingdom. To date, no homologs of LecRLKs have been reported in the genomes of fungus and human. LecRLKs are known to play roles in plant development, innate immunity [17, 20] and abiotic responses [41–43]. For example, Pi-d2, a rice G-type, and NbLecRK, a tomato L-type LecRLK, were shown to play a role in plant defense against a fungal pathogen and *Phytophthora*, respectively [9, 44]. Three tandem repeat *LecRLK* genes in Arabidopsis were shown to function redundantly to regulate abscisic acid response in seed germination [43]. Two L-type LecRLKs, LecRK-IX.1 and LecRK-IX.2, regulate phytophthora resistance and cell death in Arabidopsis [45]. A G-type LecRLK in Arabidopsis was shown to sense lipopolysaccharide, a potent microbe-associated molecular patterns from Gram-negative *Pseudomonas* and *Xanthomonas* [38]. Recently, one L-type LecRLK in Arabidopsis was shown to function as a receptor for perceiving extracellular ATP [46]. A cluster of G-type LecRLKs in rice was shown to function together to confer broad-spectrum and durable insect resistance [12]. In our study, drastic number of *PtLecRLK*s was expressed in root tissues (Fig. 7 and Table 3). Root serves as a front barrier as well as an interface for various soil microbes including bacteria, fungus, protozoa and nematodes. These root-expressed PtLecRLKs have the potential to function as receptors for perceiving signals from soil microbes. In addition, approximately 50 % of *PtLecRLK* genes were expressed at very low or undetectable level in leaf, stem and root under normal conditions (Fig. 7), implying that the expression of these *PtLecRLK*s may depend on biotic or abiotic stimuli not contained in the current tested tissues and organs or developmental stage tissues. Because no functional characterization of any PtLecRLKs has been reported to date, this represents a fruitful area for further investigation.

## Conclusions

We have reported the genome-wide identification of LecRLKs in the perennial woody model plant *Populus*. We uncovered that the LecRLK family has expanded in *Populus*. Through transcriptomics analysis, we identified a number of tissue-specific *PtLecRLK* genes. This study provides a foundation for functional characterization of this important family of receptor-like kinases.

## Additional files

Additional file 1: The sequences of primers used for RT-PCR analysis in this study. (XLSX 8 kb) Additional file 2: List of lectin domain-containing *Populus* proteins that lack the protein kinase domain. (XLSX 11 kb) Additional file 3: Comparison of different types of PtLecRLKs. (A) Amino acid sequence alignment of randomly selected PtLecRLKs from each type by ClustalW. Amino acid identity is displayed by green color above the first row in the plot. Note that the protein kinase domains at the C-terminus are highly conserved whereas lectin domains at the N-terminus are very distinct. (B) Phylogenetic tree using neighbor joining method with 1000 bootstrapping of the randomly selected PtLecRLKs. Note that three types are separated clearly. The number on branch indicates bootstrapping value of each node formation. (C) The amino acid sequence identity of examined PtLecRLKs. (PPTX 477 kb) Additional file 4: List of G-type PtLecRLKs. (XLSX 23 kb) Additional file 5: List of L-type PtLecRLKs. (XLSX 14 kb) Additional file 6: List of C-type PtLecRLK. (XLSX 9 kb) Additional file 7: List of G-, L- and C-type LecRLKs in moss, shrub, corn, soybean and *Eucalyptus*. (XLSX 18 kb) Additional file 8: Phylogenetic analysis of G- and L-type PtLecRLKs together with Arabidopsis LecRLKs using neighbor-joining method with 1000 bootstrap resampling. (A) Phylogenetic tree of G-type LecRLKs. The groups are marked by the same classification shown in Fig. 3a. (B) Phylogenetic tree of L-type LecRLKs. The groups are marked by the same classification shown in Fig. 3b. Number on the each node indicates the bootstrap value for each node formation. (TIF 7638 kb) Additional file 9: Phylogenetic tree analysis of LecRLKs from *Populus*, *Eucalyptus*, Arabidopsis and rice using neighbor joining method with 1000 bootstrap. Numbers on the node indicate the bootstrap value for each node formation. (A) Phylogenetic tree of G-type LecRLKs. Groups are marked by the same classification shown in Fig. 4a. (B) Phylogenetic tree of L-type LecRLKs. Groups are marked by the same classification shown in Fig. 4b. Convergent clade of L-type EgLecRLKs is highlighted by light red color. The blue highlighted nodes contain only rice LecRLKs. (C) Phylogenetic tree of the C-type LecRLKs. (TIF 9324 kb) Additional file 10: The amino acid sequence alignment and conserved motifs of G-type PtLecRLKs. (A) Amino acid sequence alignment via CLUSTALW. To determine conserved amino acid regions, 50 % sequence identity was used as a cutoff. Note that the amino acid sequence in the protein kinase domain is highly conserved in most G-type PtLecRLKs whereas the other domains such as S-locus glycoprotein, EGF, PAN, TM and signal peptide varied. Highly conserved motifs are boxed with labels a, b, c, d and e. The red arrows indicate truncated bulb lectin domains. The blue arrows indicate the truncated protein kinase domains. (B) The conserved motif sequences marked on panel A. Sequence logo was generated from the consensus amino acid sequence over 50 % sequence identity. (PPTX 3107 kb) Additional file 11: The amino acid sequence alignment and conserved motifs of L-type PtLecRLKs. (A) Amino acid sequence alignment via CLUSTALW. Amino acid sequence identity of 50 % was used to determine cluster of protein domain and phylogenetic node of 50 L-type PtLecRLKs. The red arrows indicate truncated legume lectin domains. The blue arrows indicate the truncated protein kinase domains. (B) The conserved motifs marked in panel A. Sequence logo was generated from the consensus amino acid sequence over 50 % sequence identity. (PPTX 3150 kb) Additional file 12: Transcript level of G-type *PtLecRLK* genes in 24 different datasets from the *Populus* Gene Atlas Study. RNA-seq data were collected from the *Populus* Gene Atlas Study in Phytozome v11.0 (http://phytozome.jgi.doe.gov/pz/portal.html). The transcript levels for each gene were expressed as FPKM. The excel sheet labeled as “whole_set” contains the original FPKM values from Gene Atlas. The data of four different tissues under standard condition are sorted in the data sheet labeled as “standard”. The group number is listed in the first column of standard datasheet based on Fig. 3 phylogenetic tree analysis result. (XLSX 62 kb) Additional file 13: Transcript level of L-type *PtLecRLK* genes in 24 different datasets from the *Populus* Gene Atlas Study. RNA-seq data were collected from the *Populus* Gene Atlas Study in Phytozome v11.0 (http://phytozome.jgi.doe.gov/pz/portal.html). The transcript levels for each gene were expressed as FPKM. The sheet labeled as “whole_set” contains the original FPKM values from Gene Atlas. The data of four different tissues under standard condition are sorted in the data sheet labeled as “standard”. The group number is listed in the first column of standard datasheet based on Fig. 3 phylogenetic tree analysis result. (XLSX 24 kb) Additional file 14: Transcript level of C-type *PtLecRLK* gene in 24 different datasets from the *Populus* Gene Atlas Study. RNA-seq data were collected from the *Populus* Gene Atlas Study in Phytozome v11.0 (http://phytozome.jgi.doe.gov/pz/portal.html). The transcript level was expressed as FPKM. The sheet labeled as “whole_set” contains the original FPKM values from Gene Atlas. The data of four different tissues under standard condition are sorted in the data sheet labeled as “standard”. (XLSX 10 kb) Additional file 15: List of *PtLecRLK* genes expressed specifically in roots. RNA-seq data were collected from the *Populus* Gene Atlas Study in Phytozome v11.0 (http://phytozome.jgi.doe.gov/pz/portal.html). (XLSX 12 kb) Additional file 16: Expression patterns of *PtLecRLK* genes in the reproductive tissues and organs. RNA-seq data were collected from the *Populus* Gene Atlas Study in Phytozome v11.0 (http://phytozome.jgi.doe.gov/pz/portal.html). The FPKM value of three female genotypes and three male genotypes were used to generate the heatmaps. The tissue specificity of gene expression was determined by the comparison of FPKM value ≥ 1 in a given plant versus FPKM < 1 in plants with opposite sex. Three different zones were categorized (Female: transcript only detected in at least one female plant but not in any male plants; Male: transcript only detected in at least one male plant but not in any female plants; Both: transcript detected in both female and male plants). (A) The expression patterns of the G-type *PtLecRLK* genes. (B) The expression patterns of the L-type *PtLecRLK* genes. (C) The expression pattern of the C-type *PtLecRLK* gene. (PPTX 9960 kb) Additional file 17: List of *PtLecRLK* genes expressed in the female and male reproductive tissues/organs. RNA-seq data were collected from the *Populus* Gene Atlas Study in Phytozome v11.0 (http://phytozome.jgi.doe.gov/pz/portal.html). (XLSX 24 kb) Additional file 18: The distribution of number of PtLecRLKs from each group in different tissues and organs. (DOCX 48 kb) Additional file 19: Diagrams of protein domain architecture of lectin domain-containing proteins that lack the protein kinase domain in *Populus*. (PPTX 63 kb)

## Abbreviations

EGF domain: Epidermal growth factor (EGF) domain
FPKM: Fragments per kilobase of transcript per million mapped reads
LecRLK: Lectin receptor-like kinase
PAN domain: Plasminogen/apple/nematode (PAN) domain
RLK: Receptor like-kinase
TM: Transmembrane domain
