# Supplementary material for: Genome-wide analysis of lectin receptor-like kinases in Populus
Source: BMC Genomics. 2016 Sep 1;17(1):699. doi: 10.1186/s12864-016-3026-2 (PMC5007699; doi:10.1186/s12864-016-3026-2)
Supplement: Additional file 3: — Comparison of different types of PtLecRLKs. (A) Amino acid sequence alignment of randomly selected PtLecRLKs from each type by ClustalW. Amino acid identity is displayed by green color above the first row in the plot. Note that the protein kinase domains at the C-terminus are highly conserved whereas lectin domains at the N-terminus are very distinct. (B) Phylogenetic tree using neighbor joining method with 1000 bootstrapping of the randomly selected PtLecRLKs. Note that three types are separated clearly. The number on branch indicates bootstrapping value of each node formation. (C) The amino acid sequence identity of examined PtLecRLKs. (PPTX 477 kb) [file 12864_2016_3026_MOESM3_ESM.pptx]

## Slide 1
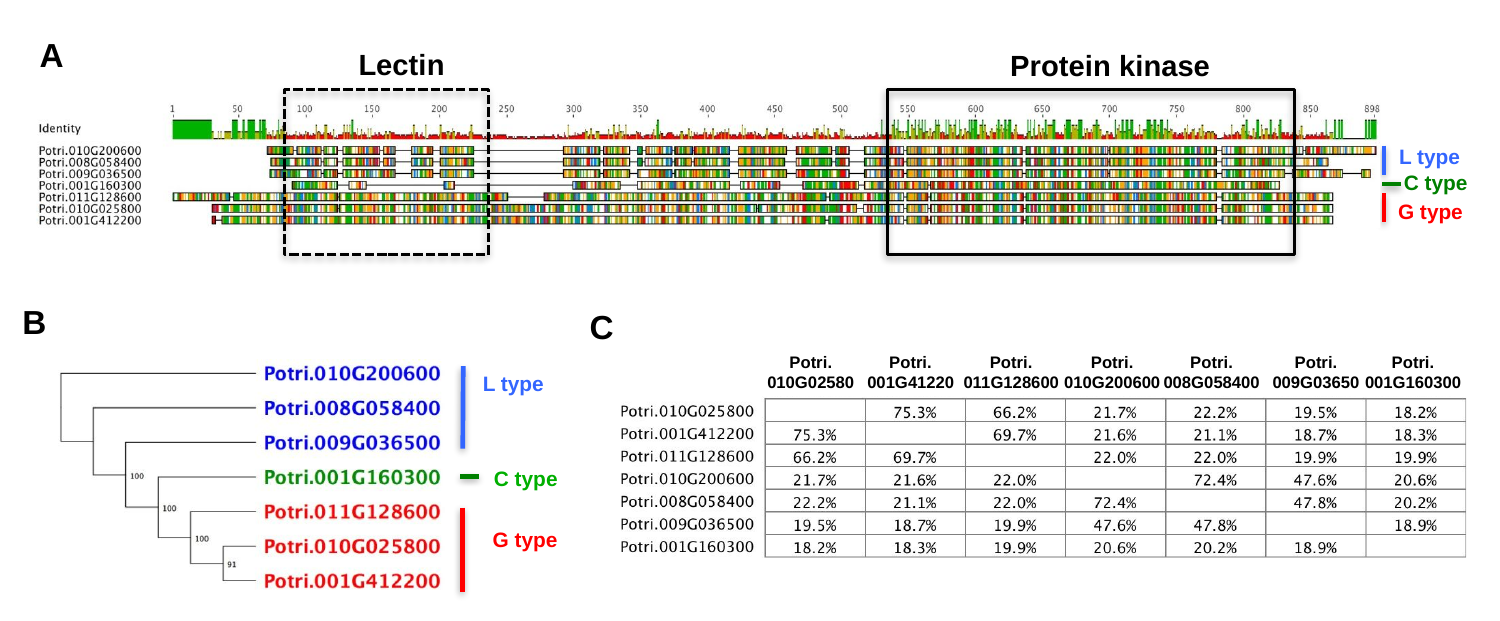

A
Lectin
Protein kinase
L type
C type
G type
B
C
Potri.
010G025800
Potri.
001G412200
Potri.
011G128600
Potri.
010G200600
Potri.
008G058400
Potri.
009G036500
Potri.
001G160300
L type
C type
G type
