# Supplementary figures and images for: Genome-wide analysis of lectin receptor-like kinases in Populus
Source: BMC Genomics. 2016 Sep 1;17(1):699. doi: 10.1186/s12864-016-3026-2 (PMC5007699; doi:10.1186/s12864-016-3026-2)

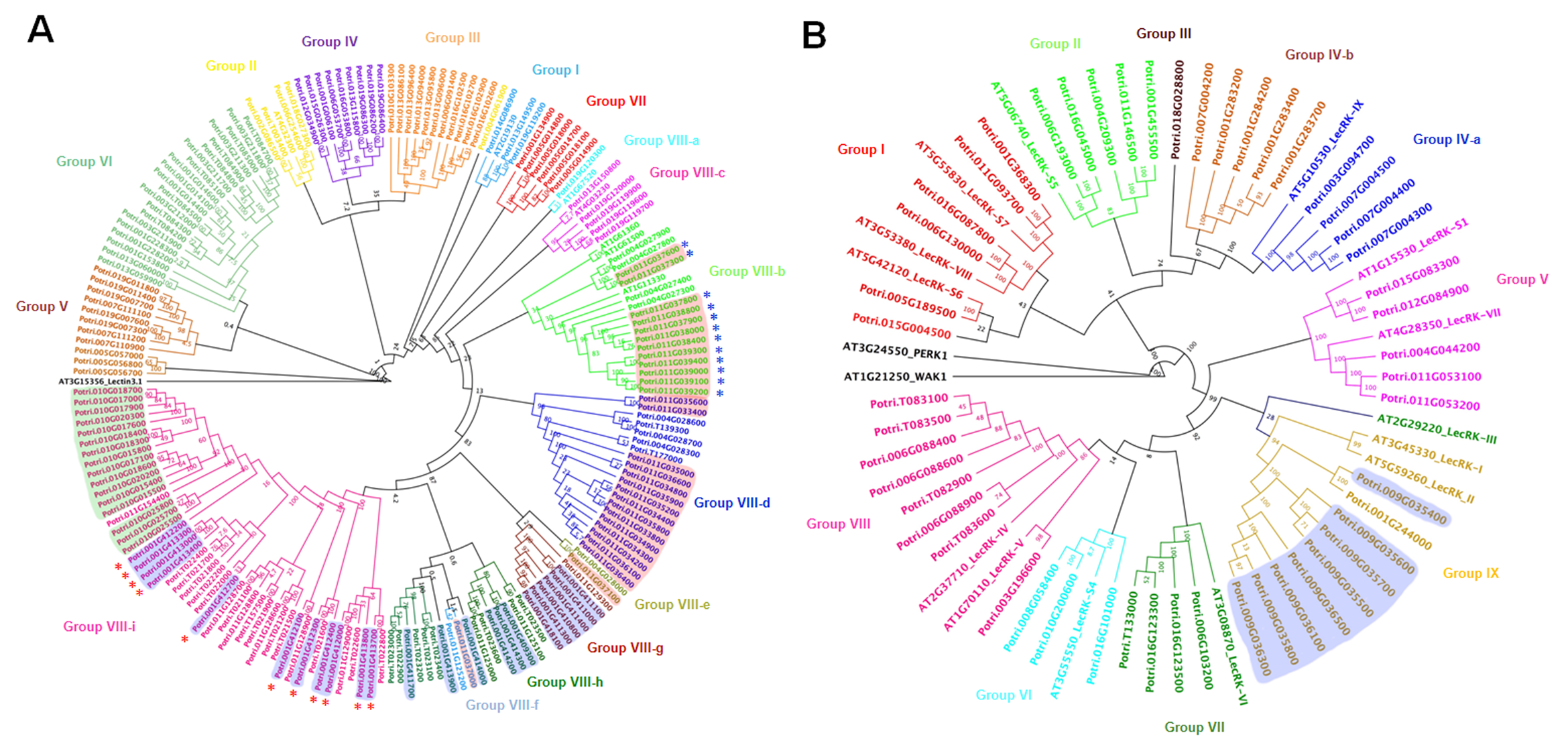

Supplement: Additional file 8: — Phylogenetic analysis of G- and L-type PtLecRLKs together with Arabidopsis LecRLKs using neighbor-joining method with 1000 bootstrap resampling. (A) Phylogenetic tree of G-type LecRLKs. The groups are marked by the same classification shown in Fig. 3a. (B) Phylogenetic tree of L-type LecRLKs. The groups are marked by the same classification shown in Fig. 3b. Number on the each node indicates the bootstrap value for each node formation. (TIF 7638 kb) [file 12864_2016_3026_MOESM8_ESM.tif]

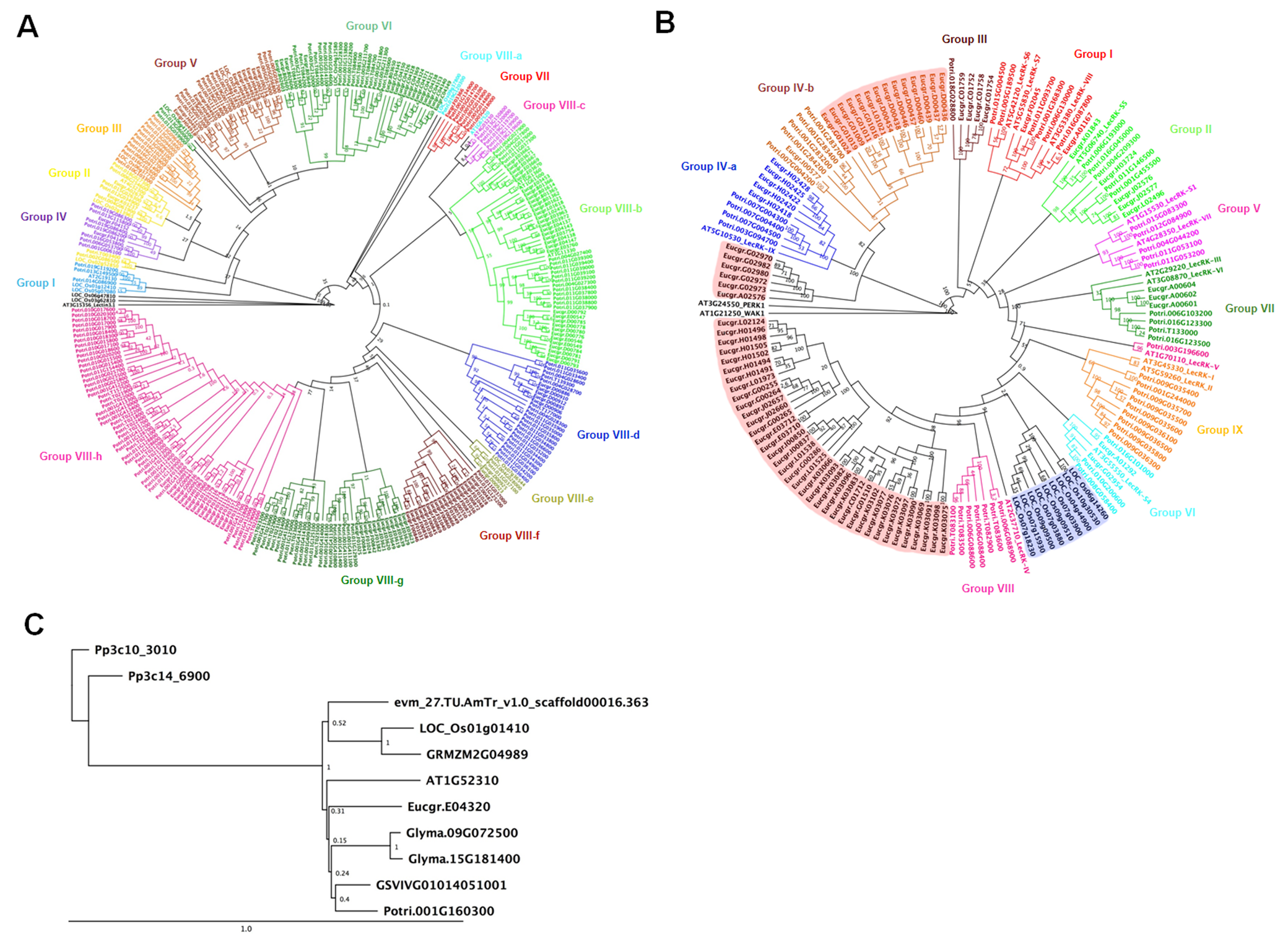

Supplement: Additional file 9: — Phylogenetic tree analysis of LecRLKs from Populus, Eucalyptus, Arabidopsis and rice using neighbor joining method with 1000 bootstrap. Numbers on the node indicate the bootstrap value for each node formation. (A) Phylogenetic tree of G-type LecRLKs. Groups are marked by the same classification shown in Fig. 4a. (B) Phylogenetic tree of L-type LecRLKs. Groups are marked by the same classification shown in Fig. 4b. Convergent clade of L-type EgLecRLKs is highlighted by light red color. The blue highlighted nodes contain only rice LecRLKs. (C) Phylogenetic tree of the C-type LecRLKs. (TIF 9324 kb) [file 12864_2016_3026_MOESM9_ESM.tif]

## Slide 1
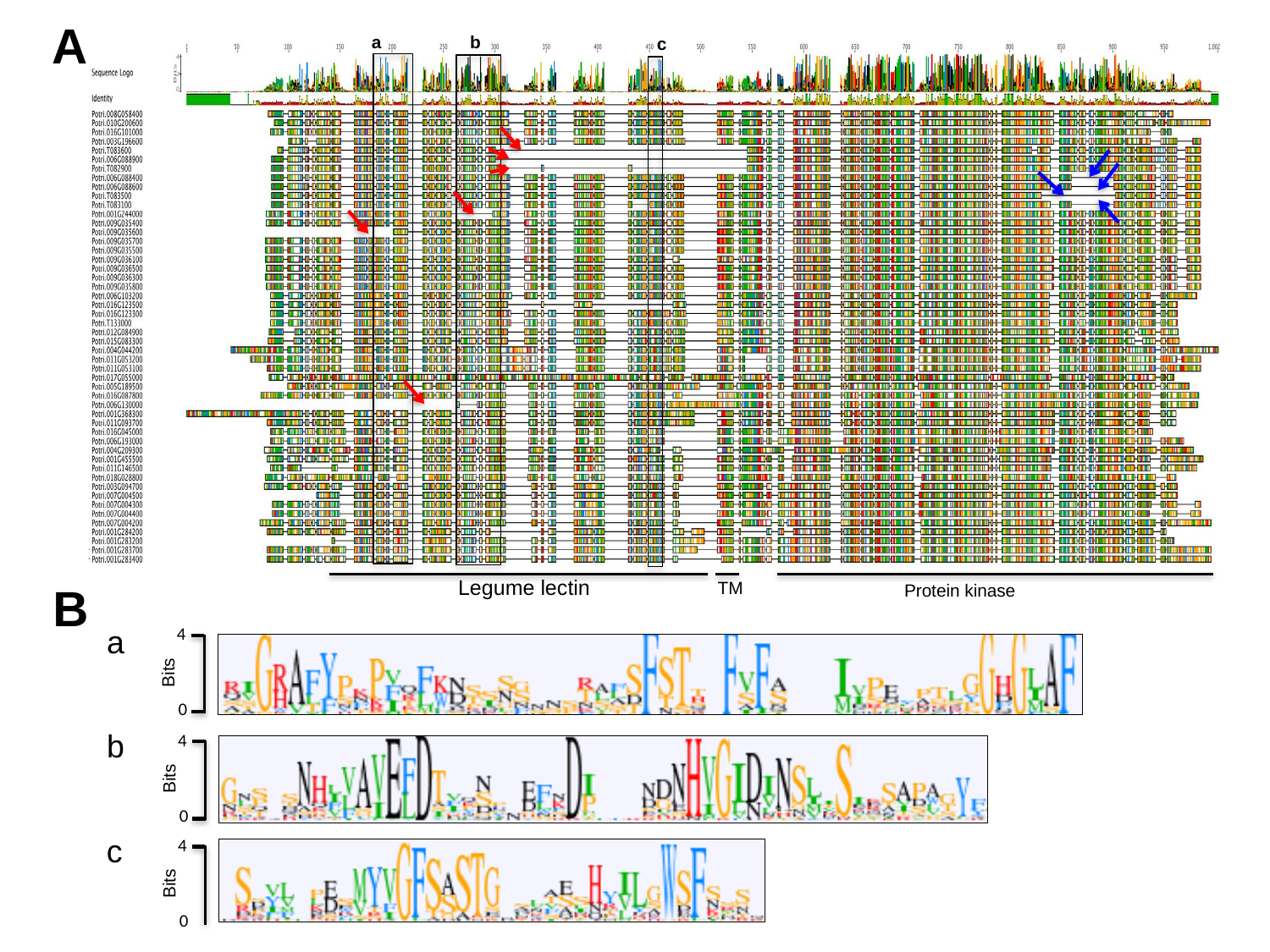

A
a
b
c
Legume lectin
TM
Protein kinase
B
a
4
Bits
0
b
4
Bits
0
c
4
Bits
0

Supplement: Additional file 11: — The amino acid sequence alignment and conserved motifs of L-type PtLecRLKs. (A) Amino acid sequence alignment via CLUSTALW. Amino acid sequence identity of 50 % was used to determine cluster of protein domain and phylogenetic node of 50 L-type PtLecRLKs. The red arrows indicate truncated legume lectin domains. The blue arrows indicate the truncated protein kinase domains. (B) The conserved motifs marked in panel A. Sequence logo was generated from the consensus amino acid sequence over 50 % sequence identity. (PPTX 3150 kb) [file 12864_2016_3026_MOESM11_ESM.pptx]
