# Supplementary material for: Genome-wide analysis of lectin receptor-like kinases in Populus
Source: BMC Genomics. 2016 Sep 1;17(1):699. doi: 10.1186/s12864-016-3026-2 (PMC5007699; doi:10.1186/s12864-016-3026-2)
Supplement: Additional file 10: — The amino acid sequence alignment and conserved motifs of G-type PtLecRLKs. (A) Amino acid sequence alignment via CLUSTALW. To determine conserved amino acid regions, 50 % sequence identity was used as a cutoff. Note that the amino acid sequence in the protein kinase domain is highly conserved in most G-type PtLecRLKs whereas the other domains such as S-locus glycoprotein, EGF, PAN, TM and signal peptide varied. Highly conserved motifs are boxed with labels a, b, c, d and e. The red arrows indicate truncated bulb lectin domains. The blue arrows indicate the truncated protein kinase domains. (B) The conserved motif sequences marked on panel A. Sequence logo was generated from the consensus amino acid sequence over 50 % sequence identity. (PPTX 3107 kb) [file 12864_2016_3026_MOESM10_ESM.pptx]

## Slide 1
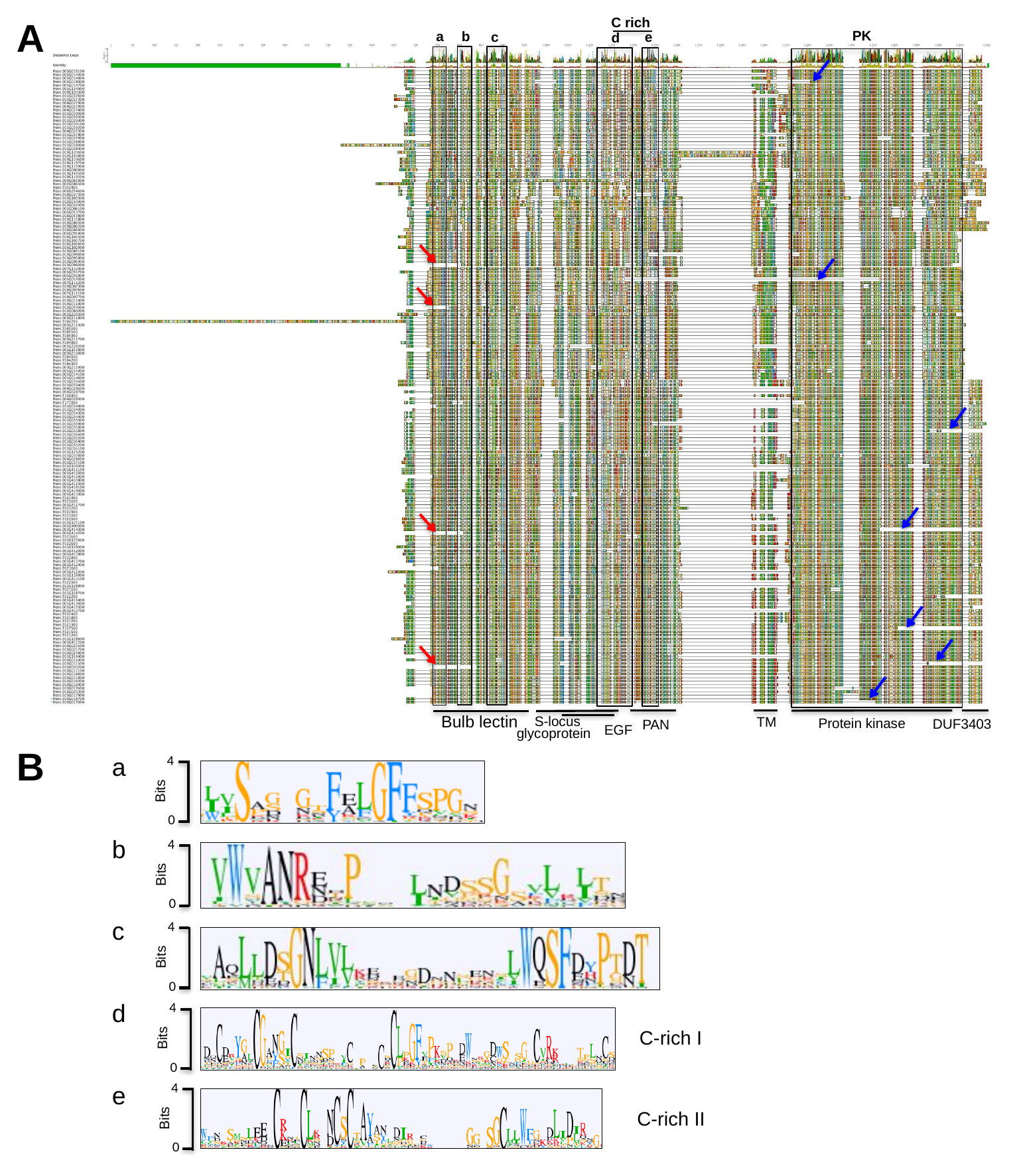

A
C rich
PK
b
a
c
e
d
Bulb lectin
TM
Protein kinase
DUF3403
PAN
S-locus
glycoprotein
EGF
B
a
4
Bits
0
b
4
Bits
0
c
4
Bits
0
d
4
C-rich I
Bits
0
4
e
C-rich II
Bits
0
