# Supplementary material for: Genome-wide analysis of lectin receptor-like kinases in Populus
Source: BMC Genomics. 2016 Sep 1;17(1):699. doi: 10.1186/s12864-016-3026-2 (PMC5007699; doi:10.1186/s12864-016-3026-2)
Supplement: Additional file 16: — Expression patterns of PtLecRLK genes in the reproductive tissues and organs. RNA-seq data were collected from the Populus Gene Atlas Study in Phytozome v11.0 (http://phytozome.jgi.doe.gov/pz/portal.html). The FPKM value of three female genotypes and three male genotypes were used to generate the heatmaps. The tissue specificity of gene expression was determined by the comparison of FPKM value ≥ 1 in a given plant versus FPKM < 1 in plants with opposite sex. Three different zones were categorized (Female: transcript only detected in at least one female plant but not in any male plants; Male: transcript only detected in at least one male plant but not in any female plants; Both: transcript detected in both female and male plants). (A) The expression patterns of the G-type PtLecRLK genes. (B) The expression patterns of the L-type PtLecRLK genes. (C) The expression pattern of the C-type PtLecRLK gene. (PPTX 9960 kb) [file 12864_2016_3026_MOESM16_ESM.pptx]

## Slide 1
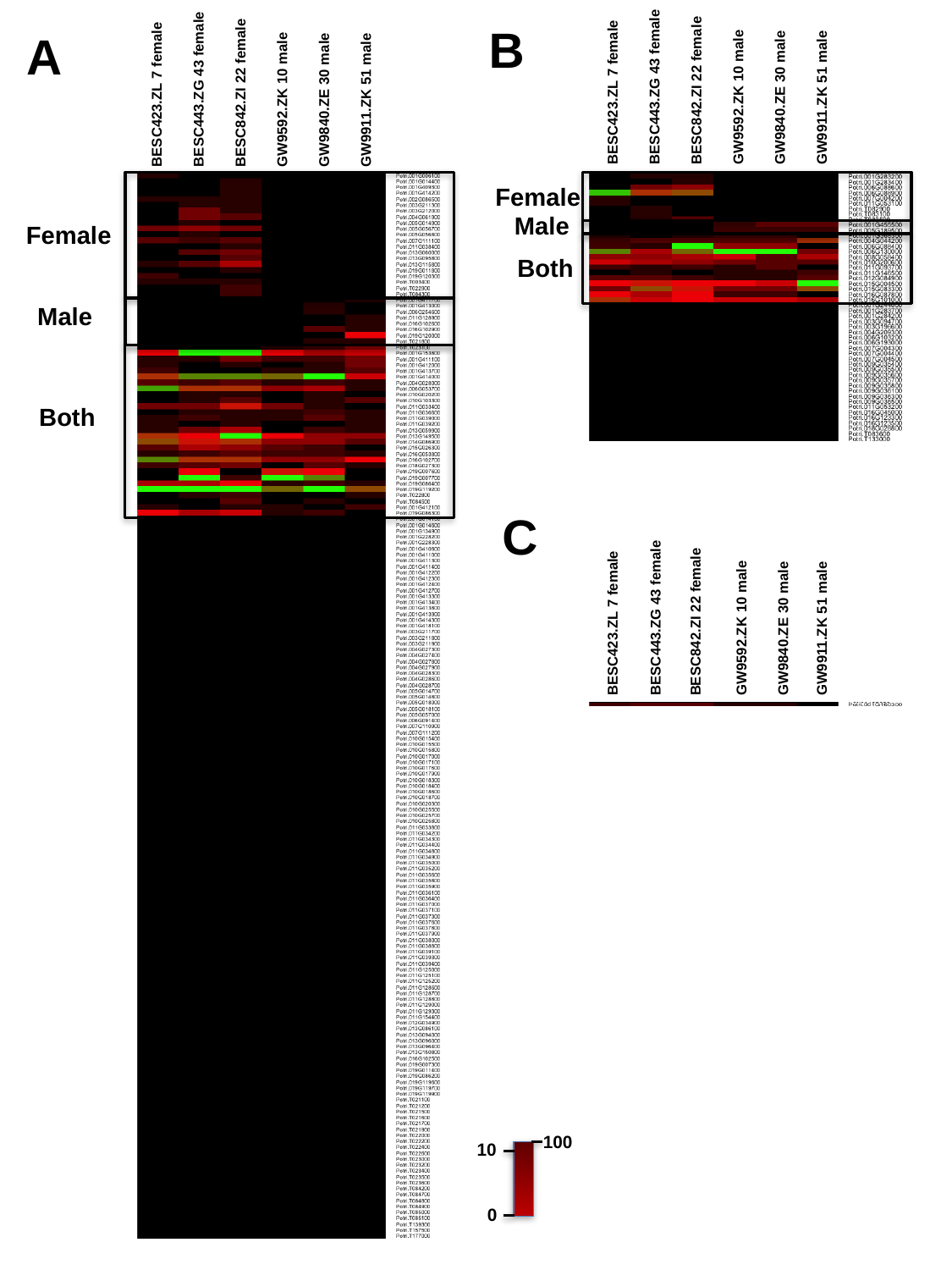

B
A
BESC443.ZG 43 female
BESC443.ZG 43 female
GW9592.ZK 10 male
GW9840.ZE 30 male
BESC423.ZL 7 female
GW9911.ZK 51 male
BESC842.ZI 22 female
GW9592.ZK 10 male
GW9840.ZE 30 male
BESC423.ZL 7 female
GW9911.ZK 51 male
BESC842.ZI 22 female
Female
Male
Female
Both
Male
Both
C
BESC443.ZG 43 female
GW9592.ZK 10 male
GW9840.ZE 30 male
BESC423.ZL 7 female
GW9911.ZK 51 male
BESC842.ZI 22 female
100
10
0
