# Supplementary material for: Genome-wide analysis of lectin receptor-like kinases in Populus
Source: BMC Genomics. 2016 Sep 1;17(1):699. doi: 10.1186/s12864-016-3026-2 (PMC5007699; doi:10.1186/s12864-016-3026-2)
Supplement: Additional file 18: — The distribution of number of PtLecRLKs from each group in different tissues and organs. (DOCX 48 kb) [file 12864_2016_3026_MOESM18_ESM.docx]

## **Additional file 18. The distribution of number of *PtLecRLK* genes from each group in different tissues and organs.**

**G-type PtLecRLKs**

**L-type PtLecRLKs**
