# Supplementary material for: Genome-wide analysis of lectin receptor-like kinases in Populus
Source: BMC Genomics. 2016 Sep 1;17(1):699. doi: 10.1186/s12864-016-3026-2 (PMC5007699; doi:10.1186/s12864-016-3026-2)
Supplement: Additional file 19: — Diagrams of protein domain architecture of lectin domain-containing proteins that lack the protein kinase domain in Populus. (PPTX 63 kb) [file 12864_2016_3026_MOESM19_ESM.pptx]

## Slide 1
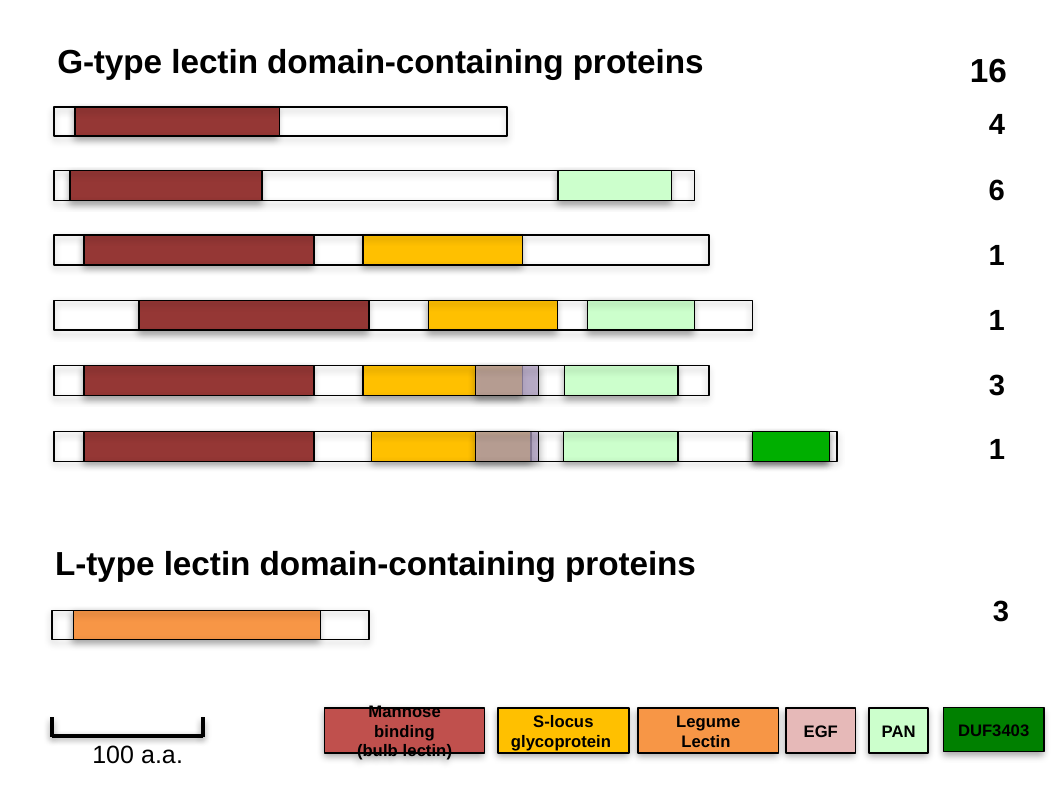

G-type lectin domain-containing proteins
16
4
6
1
1
3
1
L-type lectin domain-containing proteins
3
Mannose binding
(bulb lectin)
S-locus glycoprotein
Legume Lectin
EGF
PAN
DUF3403
100 a.a.
